# How social pharmaceutical innovations are addressing problems of availability, accessibility and affordability of drugs for rare diseases

**DOI:** 10.1186/s13023-025-04132-1

**Published:** 2025-12-08

**Authors:** Conor M. W. Douglas, Tineke Kleinhout-Vliek, Rob Hagendijk, Vololona Rabeharisoa, Wouter Boon, Fernando Aith, Claudio Cordovil Oliveira, Shir Grunebaum, Ellen Moors

**Affiliations:** 1 Department Science, Technology & Society, 307 Bethune College, York University, 4700 Keele Street, Toronto, ON M3J 1P3 Canada; 2 School of Business, University College Dublin, Belfield, Dublin 4, Ireland; 3 Faculty of Social and Behavioural Sciences, University of Amsterdam, Spui 2, Amsterdam, 1012 WX The Netherlands; 4 Sociology at the Mines ParisTech at the Université PSL, 60 Boulevard Saint Michel, Paris 75272 CEDEX 06, Paris, France; 5 Innovation and Sustainability Institute at Universiteit Utrecht, Princetonlaan 8a, Utrecht, 3584 CB The Netherlands; 6 Health Law at the University of São Paulo Public Health School and Health Law Research Center of the University of São Paulo, Av. Dr. Arnaldo, 715, São Paulo, Brazil; 7 Public Health at the Fundacao Oswaldo Cruz (ENSP/Fiocruz) National School of Public Health, Av. Brazil, 4365 - Manguinhos, Rio de Janeiro, Brazil; 8 Oxford Brookes University and Paediatric Occupational Therapist at North Middlesex University Hospital NHS Trust, Sterling Way, London, N18 1QX UK

**Keywords:** Social pharmaceutical innovation, Orphan drugs, Rare diseases, Therapeutic research and development, Social innovation, Policy, Patient organisations

## Abstract

**Background:**

The current organization of the pharmaceutical innovation system poses three major challenges for rare disease patients in terms of availability, accessibility and affordability of treatments. While some changes have emerged in the European Union to address some of these challenges, their impacts are not experienced uniformly across member states nor around the world. We have observed niche initiatives that are actively working to address those challenges within their local contexts. In a position paper in this journal, we characterized such initiatives as “social pharmaceutical innovation” (or SPIN): novel collaborations involving diverse sets of actors that break with conventional pharmaceutical innovation practices to develop interventions that address unmet societal needs of rare disease patients and that are not primarily market driven.

**Results:**

Here we report on 15 cases of SPIN across Brazil, Canada, France and the Netherlands that we studied through semi-structured qualitative interviews (*n* = 151) with players involved in those cases. Our findings show how SPIN initiatives are reconfiguring pharmaceutical innovation networks to include a wider range of actors in redistributed and differentiated roles within innovation processes. Further, we find that SPINs are associated with changes in the ways data is gathered (often in clinical contexts rather than in conventional trials), and how evidence is assembled to improve access to the treatments. Finally, we demonstrate how SPINs are providing new routes for patients to access treatments for rare diseases, often at more affordable prices.

**Conclusions:**

While promising, SPINs are not perfect solutions for rare disease patients or the broader challenges to the pharmaceutical innovation system. SPINs are specific solutions adapted to the particulars of local framing, institutions, national policy and care contexts of rare diseases, and should be developed as such. Our findings support these recommendations for SPIN: use local knowledge and expertise in crafting SPINs; develop comprehensive strategies for data governance, access and ownership; and explore new economic models to recoup investments and/or sustain future initiatives. We invite collaboration on these topics and emerging SPIN initiatives so as to support efforts at addressing challenges of availability, accessibility and affordability of treatments for rare diseases patients.

## Background & introduction

The current organization of the pharmaceutical innovation system poses major challenges for rare disease patients around the world, which commentators group into problems around availability, accessibility, and affordability of treatments. With an estimated 10,000 rare diseases [1] representing about 473 million patients globally [2], only about 4–5% of those diseases have treatments in the USA [3] and EU [4], the global leaders in terms of number of products available [5].^1^ This is market failure as companies do not see commercial profitability to develop products for these diseases, and as a result millions of patients are going untreated due to a lack of available treatments. The second challenge relates to patients’ lack of access to treatment. Newly developed treatments for rare diseases are regularly accompanied by critiques of slow approval [6, 7], reimbursement [8, 9], and the quality of evidence brought by sponsors to the approval process. While the randomized controlled trial (i.e., randomized, double-blind, placebo-controlled trials) can produce robust evidence to base regulatory and coverage decisions on, such a design -and the evidence it generates- is often mismatched with rare diseases that are characterised by small patient populations (which are often children), their life-threatening or severely debilitating nature, and common lack of comparator treatments [10, 11]. Coupled with these issues are, third, affordability problems for developed treatments. The prices for newly approved rare disease treatments are among the highest in the world, providing a real and increasing challenge to overall health system sustainability [12, 13]. Prices are generally said to be due to high costs for development and production, coupled with high risk of failure and relatively low numbers of patients.^2^

A number of important initiatives are underway to address some of these issues in jurisdictions including the European Union, such as collaboration on Health Technology Assessment (HTA) and price negotiations to increase affordability [17], and special pathways for rare disease treatments to accelerate access (e.g., the European Medicine’s Agency’s Priority Medicine’s Scheme (PRIME) [18]. However, the impacts of these initiatives are not experienced uniformly across member states nor around the world. Moreover, the current mode of pharmaceutical innovation sustains these interacting problems in the way basic and preclinical research – often initially designed and conducted in the public, charitable, and non-commercial sectors – routinely gets moved to start-ups and spin-outs/spin-offs, and subsequently transferred to larger biotech or pharmaceutical companies [19, 20]. This model, which forms the basis for the dominant biotech and pharmaceutical innovation system, is highly institutionalized, and incumbent actors have vested interests in protecting this mode and its underpinning innovation system. While research has highlighted the need to address these problems systematically [21–29], our interests is in initiatives that aim to address these challenges in a hands-on and innovative manner.

### Social pharmaceutical innovation initiatives that attempt to address problems in pharmaceutical innovation

In the pages of this journal, our consortium previously described significant changes observed in the ways treatments for rare diseases are researched, developed, and deployed. In that position paper, we characterized these changes as forms of “social pharmaceutical innovation” (or SPIN) [30], which we described to be:

novel forms of collaborative processes, programs, policies, procedures and/or designs involving diverse sets of actors that break with conventional pharmaceutical innovation practices for the production of safe, effective, and accessible interventions that address unmet societal needs of rare disease patients and that are not primarily market driven [30]. 

SPINs are set up to address the multiple, often interacting problems that exist throughout the dominant pharmaceutical innovation system, but particularly in the field of rare diseases. These problems involve the upstream processes through which research in this area is conducted and products are developed; however, they also extend to mid- and downstream patenting, regulatory, HTA, pricing, and coverage processes and procedures that are part of the broader innovation system. Importantly, we see SPINs extending beyond new products or new production processes; rather, SPINs also include processes and pathways to address issues of availability and affordability. In doing so, we shed light on the roles that can be played by governmental institutions and policy instruments – rather than exclusively researchers – thereby demonstrating that innovation is not exclusively a techno-scientific activity.

While SPINs are highly idiosyncratic, we see them as part of a broader shift in pharmaceutical research, development, and deployment. We are entering a period that features new modes of production (e.g., biomanufacturing-based production, in-hospital production, magistral preparation/compounding, platform technologies, use of big data and AI for all stages of research and development) and new products and interventions (i.e., platform-based and cell and gene therapies). This period combines both challenges and opportunities as innovations are often associated with high prices yet invite tailor-made solutions and decentralized production. We see this period as potentially increasingly characterized by “social” forms of pharmaceutical innovation: networked partnership types and heterogeneous collaboration. While SPINs garner relatively tiny amounts of investment in research, development and deployment of pharmaceuticals, and make up a small percentage of new products and initiatives, disruption and re-organization of the sector are underway that these SPINs evidence and accelerate.

This article explores how SPINs tackle problems of unmet needs and innovation system failure. We present the findings of our case studies that illustrate how SPINs are re-organizing research into more diverse and collaborative forms, transforming how data is gathered and used, and opening new routes for patient access to treatments. We focus on the following questions: What are the defining features of the investigated SPIN initiatives (Section  “Findings”)? How do SPINs function – from both a substantive and social perspective – to address the issues of treatment availability, accessibility, and affordability (Section  “Summary & discussion – addressing availability, accessibility and affordability through SPINs”)? What lessons can we learn from these initiatives to inform recommendations that support future SPINs (Section  “Conclusions, recommendations & future work”)?

## Methods

The project started with desk-based research on the current pharmaceutical innovation systems as institutionalized in our four respective countries of Brazil, Canada, France and the Netherlands. This ‘mapping’ work was instrumental in delineating the multifaceted social, economic, political, and regulatory landscapes encountered by our SPIN cases. Across the four countries, we compiled information on societal discussions on drugs for rare diseases in the media and the existence and roles of patient groups and advocacy organizations. We scoped national R&D systems in biotech and pharma sectors. We collected data on national expenditures on drugs for rare diseases (DRD), national DRD strategies, and regulatory, licensing, and HTA processes for DRD. This dense and wide-ranging background work is not presented here but provided crucial context for understanding our case studies.

Next, we established a “multiple case study” research design of fifteen case instances of social pharmaceutical innovation across our four countries (i.e., three in each of Brazil, Canada and France and six in the Netherlands). An overview of the case studies is provided in Table 1 below, with details of case studies presented in Section  ‘Findings’.

A multiple case study design (MCSD) is not the same as a comparative case study. MCSD focuses on replication logic where each case is treated as a separate experiment. In this approach the focus is on analysis of the contextual environment in which each case operates, and provision of detailed understanding of how cases operate within real-world context. A MCSD facilitates the elucidation of themes across cases and is not focused on the formal comparison of a limited number of variables across cases as in comparative case study design. Because SPINs are entrenched in very different national systems that affect the constitution of initiatives, direct comparison is impossible. What is more, not all initiatives or treatments in questions were available across our four countries. Taken together MCSD was the most appropriate research design for understanding SPINs across our four countries.

We selected cases that addressed problems of availability, accessibility, and affordability in innovative ways relative to – and within – their national context. What is innovative in one context may be rather regular in another, and what is possible in one country may not be in others. We wanted case studies to reveal innovation within (national) contexts. We therefore selected a variety of cases along the life cycle of drug research, development and deployment that sought diverse ways of addressing different kinds of problems in rare disease innovation.

Case study data collection involved qualitative research (i.e., semi-structured interviews), participation in multistakeholder meetings, document analysis (i.e., innovation policies, regulatory decisions, media articles, websites), and other background case information. Across our fifteen case studies we conducted one-hundred and fifty-one semi-structured interviews in total (Brazil *n* = 32, Canada *n* = 48, France *n* = 15 and Netherlands *n* = 48; total *n* = 151) with a range of participants involved in SPIN cases including representatives from government ministries, non-governmental institutions, pharmaceutical and biotech industry representatives, members of patient organizations, clinicians, clinical researchers, pharmacists, project leaders, policy makers, health care insurers, regulators, and funders. Initial interviewees were identified through the analysis of public documents on cases, and subsequent interviewees were identified through snowball techniques. All interviewees were recruited via email invitations that included project description, and consent forms (in countries in which this was required). Key questions driving these interviews pertained to the nature of the case study, its history, identification and roles of associated actors, description of how the case works, goals of initiative, and facilitators and barriers experienced along the way. Each national team developed their own interview questions on these shared topics in their own language, which were tailored to the specific case in question. Interviews were primarily conducted on-line, lasted between 30 and 90 min, and were recorded. Interviews were manually analysed by respective national teams for the elucidation of key themes associated with the above-mentioned lines of inquiry.

Our consortium met numerous times to discuss themes emerging from our respective case studies with the most prominent of those being: (1) SPINs in research, development & manufacturing; (2) SPINs in market access, coverage & pricing; (3) scaling-up & reproduction of SPINs. Those three themes were explored in a working paper that served as an input for our two-day event in March 2023 in Utrecht: the “Global Conference for Advancing Social Pharmaceutical Innovation”. The objectives of that event were (a) outreach of our project to those involved with SPIN type innovation, (b) community building across SPIN initiatives, and (c) validation of our case study findings. At that event, each theme was discussed by panels of experts from various institutions (i.e., social innovation initiatives, governments, hospitals, companies, public-private partnerships, patient organizations, advocacy organizations, academia) and with different national backgrounds (i.e., the Netherlands, Brazil, France, and Canada). Experts responded to our findings on these themes. After each panel discussion, all participants went to in-person or online break-out rooms. These break-out sessions consisted of structured discussions on pre-set topics derived from the presentations and panels, which varied from room to room and were chaired by researchers from our consortium. The main lessons were gathered by the Chairs and briefly presented during wrap-up plenary sessions. Participants to the sessions of the outreach event discussed our consortium’s findings and provided feedback on the challenges ahead for pharmaceutical innovation and rare diseases. The results from the event were written down and discussed during a two-day on-site project meeting in Utrecht in April 2023, which make up the findings presented here.

**Table 1:** Summary of case studies

Country / case	Treatment	Condition(S)	Kind of spin
BRAZIL − 1- *Parcerias para o Desenvolvimento Produtivo /* Parternships for Productive Development	Biosimilar development technology for taliglucerase alfa	Gaucher’s Disease Type I	Improving access and affordability
BRAZIL − 2- ANVISA Resolution 205/2017 trust pathway for expedited access	Various treatments for rare diseases	Various rare diseases	Facilitating access and increase therapeutic options (availability)
BRAZIL − 3- Pilot study of risk-sharing agreement	nusinersen (Spinraza^®^)	Spinal Muscular Atrophy (SMA) Type I	Improving access and affordability
CANADA − 1- Genomics for Rare Diseases (G4RD) data sharing platform	Various treatments for rare diseases	Various rare diseases	Diagnostic tool and foundation for various treatments for rare diseases (availability)
CANADA − 2- BioCanRx made-in-Canada hospital manufacturing	CD19 CAR T cell therapy	Particular forms of non-Hodgkin’s lymphoma or acute lymphoblastic leukemia	Improving access and affordability
CANADA − 3- Canadian Fabry Disease Initiative a post-market coverage with evidence agreement	agalsidase alfa (Replagal^®^) and agalsidase beta (Fabrazyme^®^)	Fabry’s disease	Facilitating availability, access and affordability
FRANCE − 1- Public-Private-Patient collaboration in gene therapy development	lenadogene nolparvovec gene therapy (Lumevoq^®^)	Leber’s hereditary optic neuropathy	Creating new treatment (i.e., increasing number of available treatments)
FRANCE − 2- Patient-driven licensing agreement & Public-Private-Patient collaboration in gene therapy development	GNT0004 experimental gene therapy	Duchenne Muscular Dystrophy	Creating new treatment (i.e., availability) and ensuring, access to the therapy
FRANCE − 3- Public-Private-Patient collaboration in gene therapy development	onasemnogene abeparvovec (Zolgensma^®^)	Spinal Muscular Atrophy (SMA)	Accelerating access and facilitating debates on affordability
NETHERLANDS − 1- ‘Potentially Promising Care’ subsidy for ‘in-house’ hospital manufacturing	CAR T cell therapy	Relapsed or refractory diffuse large B-cell lymphoma	Facilitating access to treatment
NETHERLANDS − 2- CUREiHUS research protocol	eculizumab (Soliris^®^)	Atypical hemolytic uremic syndrome (aHUS)	Facilitating access and improving affordability
NETHERLANDS − 3- myTomorrows clinical trials participation platform	Numerous	Numerous	Facilitating access to treatment
NETHERLANDS − 4- Magistral preparation / pharmacist compounding	chenodeoxycholic acid and mexiletine	cerebrotendinous xanthomathosis (CTX) and myotonia patients	Facilitating access and improving affordability
NETHERLANDS − 5- Patient One collaboration for fairly priced and accessible medicine	Sustained (slow)-release cysteamine bitartrate	cystinosis	Facilitating availability, accessibility, and affordability
NETHERLANDS − 6- LIDOPAN repurposing study	lidocaine	Pancreatic cancer	Facilitating availability and accessibility to treatment

## Findings

The analysis of the case study data and the validation during the outreach event led to a focus on three key features of SPINs: (1) their expanded collaborative nature and re-organized roles of involved players; (2) their transforming modes of data collection and use in innovation processes; and (3) their provision of novel routes to treatments that patients can travel for access. We have selected cases to illustrate broader points observed.

### Reconfiguring innovation networks in pharmaceutical innovation

A key feature of the studied SPIN cases was their re-organization and associated redefinition of who is – and should be – involved in addressing the sectors’ innovation problems. SPIN cases show how a broader involvement of different actor groups is organized and how redistribution of innovation work can and does occur. While the industry is primarily motivated by return on investment [31], these more diverse collaborations result in broadening interests pursuant to the development and availability of more treatments.

#### More diverse sets of actors, more collaborative forms of partnership

SPINs are first and foremost partnerships that require a broader range of players. We saw this at work in the development of the Lumevoq© gene therapy for Leber’s hereditary optic neuropathy. Patient organization AFM (French Muscular Dystrophy Association) is a major actor in rare disease research and development (R&D) in France and in Europe. In 2004, AFM’s lab Généthon collaborated with a French university-hospital institute called *Institut de la Vision* in the development of a vector for a gene therapy targeting the eye. AFM also supported the creation of a biotech spin-off called GenSight in 2012, again with the university-hospital institute, and supported through a venture capital fund of the French public investment bank and the AFM. Rather than discoveries being transferred from public institutions into the private sector, here we see long-term collaborative research and development aimed at setting up and consolidating a French research, development, and manufacturing environment conducive for gene therapies to foster availability close to patients suffering from rare diseases. This broad range of actors played critical roles in supporting the development of a therapy for a condition that had limited treatment options. At the time of study, GenSight Biologics was applying for market approval at the European Medicines Agency (EMA), and continued testing on different sub-populations of patients to expand the availability of treatments for this very rare disease.

The Dutch case study of Patient One also demonstrates this feature of broader collaboration. Patient One was established in 2017 as an experimental public-private collaboration, involving a pharmaceutical company, social investors, development experts, academics, patient representatives, and clinical experts. The formal agreement between these parties is based on the Fair Medicine Charter, of which the development was funded by the Dutch Ministry of Health [32]. Patient One aims to develop a slow-release, “accessibly priced” version of a pre-existing treatment for cystinosis, a rare genetic disorder where the amino acid cystine builds up in the body’s cells, causing crystals to form and damage organs, particularly the kidneys and eyes, affecting about 1 in 100,000 [33]. As the currently available medicine has to be taken every six hours exactly, the slow-release version is easier for patients and their caregivers and “accessibly priced”. These cases show that broader forms of collaboration mobilize diverse actors not to maximize financial returns, but to expand treatment access and affordability by investing in local infrastructures, public–private partnerships, and patient-centered goals.

#### More diverse sets of innovation activities

Our study also highlights changes in the content of collaborations between different players. In another case, French AFM collaborated with American biotech Sarepta Therapeutics and YposKesi (i.e., AFM’s contract development and manufacturing organization) in the development of GNT0004, an experimental gene therapy for Duchenne Muscular Dystrophy (DMD). In this partnership, AFM contributed the gene therapy vector developed by its lab Généthon, and Sarepta Therapeutics, a technology called exon-skipping. The partners signed a collaborative licensing agreement, released in 2020, which stipulated that: (i) Généthon was responsible for commercializing the GNT0004 product in Europe (outside England) and Sarepta in the rest of the world; and (ii) YposKesi had been entrusted with production. At the time of study, clinical trials were underway. AFM’s goal was to act as a market operator to increase the availability of the product so that French and European patients could access the therapy through a novel market design: the involved organizations thus engaged in activities typically in the domain of commercial actors.

#### Redistributing roles and capabilities

Across many of our SPIN cases, public sector researchers and practitioners become more and more directly involved in product development and manufacturing to improve treatments, availability, and accessibility of medicines. For instance, in the Netherlands, chenodeoxycholic acid and mexiletine had long been prescribed off-label to cerebrotendinous xanthomathosis (CTX) and myotonia patients, respectively. However, controversies erupted when the price of the commercially available treatments increased substantially for these rare disease patients specifically, and suspicions arose of some companies gaming the regulatory system to raise their profits. Following these sharp price increases, access to the treatment came under pressure. As a result, pharmacists collaborated with medical specialists in the production, or compounding, of these treatments (also known as magistral preparation). Following intense public debate and commercial pushback between 2012 and 2021, the Dutch Healthcare Authority (NZa) extended the regulatory conditions under which magistral preparation is allowed. The two treatments in question are now magisterially produced at the pharmacy of Amsterdam Medical Centre (AMC) and no longer exclusively by pharmaceutical industrial actors. Dutch pharmacies are now allowed to sell to other pharmacies magisterially produced medicines that have been placed on a closed list of products that received negative coverage advice [34].

Other cases with public actors engaging in manufacturing activities include the Canadian Biotherapeutics for Cancer Research (BioCanRx) network’s Canadian-Led Immunotherapies in Cancer (CLIC) trials program and the ‘Potentially Promising Care’ subsidy to the Dutch University Medical Centre Groningen (the latter discussed in more detail below). Both cases are examples of ‘in-house’ development and production of CAR T-cell therapy. Launched in 2016, the CLIC trials program produced an entirely made-in-Canada treatment to reduce vein-to-vein time. Furthermore, BioCanRx and the CLIC program proved that all aspects of treatment production for a set of rare cancer variants could be achieved entirely within an academic setting [35, 36], thereby making treatments more accessible to a wider set of patients. Manufacturing entirely within the public sector not only worked to build Canadian capacity but also proved that advanced biotherapies could be produced entirely outside of industry. Additionally, their treatment targeted a broader indication thereby providing improved availability of care. Their trial has treated ninety patients and established a research and manufacturing platform for further CAR T treatment development.

### Changing the ways data is gathered, and evidence is assembled

Our cases demonstrate the critical role of data and evidence in pharmaceutical innovation. SPIN initiatives work to prepare and arrange new options for collecting and analysing data and generating evidence for treatments for specific conditions, thereby improving accessibility, affordability and availability.

#### Articulating and gathering data on diagnosis, clinical efficacy, and efficiency of the treatment differently

One key problem of rare diseases is the near-impossibility of achieving traditional double-blind placebo-controlled clinical trials. Our case studies highlight diverse attempts to generate different kinds of data and evidence. For instance, the Dutch CUREiHUS case shows how access to a rare disease treatment can be facilitated when coupled with a data collection research protocol. The CUREiHUS research emerged as a collaboration between clinicians and researchers at Radboud University Nijmegen and several patient groups to pre-empt a possible negative coverage decision on eculizumab (Soliris^®^) by the Dutch National Health Care Institute. The partnership proposed to admit eculizumab to the basic health insurance package to treat aHUS patients, but *only* when administered as taking part in this special research protocol. This protocol involved the strict application of treatment start-stop criteria informed by (real-world) data gathered by clinical researchers in patient monitoring and storing these data in a tailor-made database. Upon diagnosis, patients would only receive three months of treatment, followed by longitudinal study of the effects of the treatment in a closely monitored way [37]. Further treatment would only be administered in case their situation worsened. The CUREiHUS protocol is thought to have saved 60 + million euros, provided access to a treatment that might not have been covered, and the criteria have been transferred to the Dutch treatment guidelines as of 2023 [37].

#### Taking hold of the collection, ownership, and use of clinical evidence

In contrast to siloed and industry-dominant data production through clinical trials, some SPIN initiatives rely on different actors to collect data. In these SPINs, clinicians and clinical research settings provide treatments to patients that would otherwise be inaccessible. This was not only visible in the CUREiHUS research case, but also in the Canadian Fabry Disease Initiative (CFDI) launched as a partnership to collect further evidence of the efficacy and cost-effectiveness of then-novel enzyme replacement therapies (ERT) in a real-world setting. While Health Canada had granted Notice of Compliance (i.e., market authorization) for agalsidase alfa (Replagal^®^) and agalsidase beta (Fabrazyme^®^) for the treatment of Fabry diseases, a subsequent HTA coverage recommendation for the ERTs in 2005 advised to “not cover at that price” [38, 39]. In response, the sponsor companies discontinued access for Canadian patients who had been receiving ERT treatments from 2001 to 2004 through clinical trials or on a compassionate use basis. In a scramble to ensure treatment continuity and provide access, the CFDI launched as a post-market clinical research study collaboration between the sponsor companies, the federal and provincial Ministries of Health, and a team of clinical research experts in Fabry from across several Canadian provinces. All Canada-based patients that were prescribed the ERTs were enrolled in the CFDI, providing access to treatment while collecting ‘real world’ efficacy data [40]. Over the next twelve years, the university hospital leading CFDI took hold of the production, storage, and use of clinical evidence in a publicly owned data infrastructure, facilitated access to ERT to over six hundred patients, and constructed a treatment protocol now adopted across Canadian provincial health systems [40, 41].

We also see this taking hold of data production in the Dutch LIDOPAN study, a collaborative, patient-initiated clinical trial to establish the value of lidocaine (i.e., a common local anaesthetic used by in a wide variety of procedures like suturing wounds in emergency departments or for dental procedures) for pancreatic cancer pain relief. According to patient organization Inspire2Live, pain in cancer patients is often ignored, as survival is deemed the more important outcome measure. As pancreatic cancer is often considered one of the most painful cancers, Inspire2Live collaborated with the Dutch Pancreatic Cancer Group (DPCG) and hospital-based researchers at the Amsterdam Medical Centre (AMC) for the LIDOPAN study [42]. Here the Inspire2Live patient group moved from a position of exclusion from the treatment process to the initiator of the study and became the driver of the collaboration (with data collection again by clinical researchers).

#### Creating disease-oriented instead of primarily drug-oriented registries

Market authorization of a drug for a specified medical treatment is based on the data about the properties of the drug, its efficacy and safety that are owned by the applicants – generally a private company. Yet in recent years, commentators have stressed the importance of developing public sector registries that serve both diagnosis and treatment of patients as well as clinical research [43, 44], in other words both pre and post-market uses. The European Reference Networks provide an early example of this knowledge exchange [45]. Disease-based registries are broader data infrastructures used for facilitating gene discovery, improving diagnostic processes, and better understanding disease evolution, among other applications. The Canadian Genomics4RareDiseases (G4RD), launched in 2022 by the Care4Rare consortium [46, 47], provides a good case example of such a broader data infrastructure. G4RD works to secure access to genomic data from across provincial healthcare systems to pool it for diagnosis, gene discovery, and treatment development. G4RD conceptualizes itself as a “data lake” for rare disease researchers, where genomic and health-related data can be “dumped” into computerized cloud storage and “pumped” out to provide new insight by way of data mining and AI capabilities [48]. G4RD now links over 3,000 cases with diverse clinical and multi-omic data from across Canada and connects to other international databases for gene matchmaking potential. A patient re-contact registry allows patients to consent to sharing their clinical data produced in provincial health systems by new genomics screening programs, as well as other research endeavours. The long-term goal is to establish a federated platform where data from diagnostic sequencing remains under the jurisdiction of provinces but is more widely accessible for diagnostic and research purposes by way of a tiered data governance strategy [47].

### Providing new routes for patients to access treatments for rare diseases

SPIN initiatives may seek to improve the attainability of existing options instead of generating new medicines and treatments for rare diseases. Indeed, our case studies show new routes and connections to quicker, easier, and more affordable access to already-developed treatments.

#### Accelerating access to treatments

Several studied SPINs relied on early access paths to innovative treatments, which ranged from conditional approvals, initiatives that facilitated expedited regulatory reviews, as well as expanded access to clinical trials. During the 2000s, the AFM and French public research institutions developed the vector used in gene therapy onasemnogene abeparvovec (Zolgensma^®^) for Spinal Muscular Atrophy (SMA). An American biotech start-up called AveXis conducted the clinical trials. To secure access for French (and European) patients, the AFM arranged a licensing agreement for clinical trials to be conducted by AveXis at a university-hospital institute created by the AFM and co-operated with French public research organizations. AveXis was later acquired by Novartis which put the therapy on the American market in 2019, and got a conditional marketing authorization from EMA in 2020. The AFM, its sister organizations, and collaborating clinicians asked for early access from the French Medicines Agency while waiting for additional clinical trials [49] and pricing negotiations between Novartis and the French public authorities. The list price for the treatment ($2.1 million USD) raised controversy on the affordability of the treatment. To address this issue, the AFM is engaging in discussions with regulatory bodies and various stakeholders in orphan drug markets, and is actively participating to public debates on drug pricing mechanisms, and on the limits of the exclusively for-profit economic model of pharmaceutical innovation.

The Brazilian Health Surveillance Agency’s (ANVISA) Resolution 205/2017 provides a second example of accelerated access. This special procedure for the approval of clinical trials, certification of manufacturing practices, and registration of new drugs for the treatment of rare diseases came into effect in 2017 through cooperation between ANVISA, representatives from the pharmaceutical industry, and civil society. It allowed product sponsors to submit preliminary data for regulatory approval while committing to provide final data with the completion of clinical trials. In addition, the Brazilian National Regulatory Agency (NRA) may rely on the assessment of other regulatory agencies for their own decisions. By combining rolling submission of clinical trial data and other regulators’ assessments, the Resolution cuts average processing times for regulatory applications for biological products for rare diseases from 351 to 170 days and from 297 to 196 days for new synthetic drugs for rare diseases [50, 51]. Brazil has also experienced faster access to treatment, flexibility for trial recruitment, and lower registration costs [50, 51].

Finally, the Dutch myTomorrows case demonstrates a modified pathway through which patients access products currently in clinical trials. MyTomorrows is a two-sided platform (likened to a ‘booking.com’) to connect patients to pharmaceutical companies’ Expanded Access Programmes. Many patients cannot take part in ongoing clinical trials because they are ineligible due to, for instance, their country of residence or specific comorbidities. Physicians and small- or medium-sized companies tend to initiate contact concerning a specific case or treatment. After the patient is administered the medicinal product, myTomorrows collects real-world data for the company, thus reinforcing the role data plays in enabling some SPIN initiatives [52]. The company’s website states that they have helped over 5,000 patients, and the database currently holds 300,000 + clinical research studies conducted in over 175 countries.

#### Bringing treatments close(r) to patients

Another way that SPINs provide access for patients is localizing manufacturing: bringing treatment manufacturing closer to patients. We saw this at work in the Brazil’s *Parcerias para o Desenvolvimento Produtivo* (which translates to Partnerships for Productive Development, or PDP) between Pfizer, Protalix Biotherapeutics, and the Secretariat of Science, Technology, and Strategic Inputs (SCTIE) at the Ministry of Health to transfer biosimilar development technology for taliglucerase alfa to treat Gaucher’s Disease Type I. Brazilian Partnerships for Productive Development are technology transfer agreements from private companies to public institutions to foster local production of specific health products thereby reducing the country’s dependence on foreign producers.^3^ Established in 2013, the PDP allowed Brazil’s Institute of Technology in Immunobiologicals (Bio-Manguinhos/Fiocruz) to produce taliglucerase alfa and analyse the costs and benefits of the partnership over a five-year period, with an eye to alternative systems for the production and supply of medicines for the Unified Health System (i.e., the publicly funded universal healthcare system that guarantees free access to health services for all residents of Brazil, which is also known as SUS). While the initiative was cut short by the Ministry of Health due to regulatory and technological transfer issues, the PDP set the basis for acquisition of treatments for rare diseases at better prices for SUS. The PDP model of SPIN is still at work in Brazil (Ordinance GM/MS No. 4,472/2024), and as of 2019, Bio-Manguinhos was the sole public laboratory supplying taliglucerase alfa [53].

Other cases demonstrated how local clinical trials for patients facilitated access to treatment and how the localization of trials was coupled with point-of-care manufacturing of the treatment. In 2020, the Dutch National Health Care Institute (ZIN), together with the Dutch health care funder (ZonMw), granted a 30-million euro ‘Potentially Promising Care’ subsidy to the University Medical Centre Groningen and several other university hospitals for point-of-care ‘in-house’ production of CAR T-cell therapy. Once produced, this therapy would be evaluated against the commercially available comparator product, which takes over a month to produce, while most patients only have a few months to live. This ‘in-house’ CAR T-cell therapy is to be provided under the EMA’s hospital exemption pathway [54]. The goal was not primarily to challenge the pricing of the comparator product but to show that alternative manufacturing was possible. Not only did this seek to speed up the time it took for treatment development as patients’ T-cells are needed to develop the treatment, but it would also hopefully lead to quicker delivery as treatment did not have to be frozen and shipped to and from abroad. In both this Dutch case and the BioCanRx CLIC-01 trial that built a CAR T-cell treatment entirely in Canada and entirely within the public sector [35], treatment development was localized to the point of care. In the same vein, all French case studies focused on how the AFM sought to contribute to the structuring of an entire public-private industrial sector of advanced biotherapies close to French patients, but also to develop affordable treatments (though Zolgensma^®^ unfortunately failed in this case).

#### Paying and pricing differently

New routes for patient access to treatments are also characterized by changes in how treatments are covered and paid for. Rather than the usual HTA procedures (through which ‘all or nothing’ coverage decisions are made based on conventional evidentiary requirements), SPINs are demonstrating how to account for the uncertainty on treatment performance as well as contain high prices. Such initiatives take many forms, such as managed access, pay-for-performance, and risk-sharing agreements (RSA) [55–58]. An example of the latter is provided by our Brazilian case study of the Ministry of Health’s pilot study for conditional coverage of nusinersen (Spinraza^®^) in 2019 to treat Spinal Muscular Atrophy (SMA) 5q aiming types II e III (i.e., Ordinance GM/MS nº 1.297/2019) and its potential incorporation into SUS. Treatment would have been unavailable due to high costs, so the RSA was meant to address clinical uncertainty of the treatment while making it accessible to patients. Payment to pharmaceutical company *Biogen Brasil* would have been conditional upon the treatment meeting pre-set clinical outcomes. Patient monitoring was carried out at reference centres coordinated by a research institution, and additional evidence would have been produced and submitted to the National Committee for Technology Incorporation into the SUS (also known as *Conitec*) within three years for reassessment. Conitec is a permanent collegiate body, part of the regulatory structure of the Ministry of Health, which advises on adopting new health technologies in the SUS. It evaluates scientific evidence, cost-effectiveness, budget impact, and social value. Unfortunately, in 2020, this pilot study was discontinued. Eventually the SUS expanded the use of nusinersen to include patients with SMA type 2 in 2021, and RSAs have been set up for other rare disease treatments [59]. Other SPIN case studies (i.e., the Canadian Fabry Disease Initiative and the Dutch CUREiHUS treatment protocol) have explored similar procedures to simultaneously facilitate access and contain costs. At the same time, yet other SPIN case studies, particularly the French, demonstrate how the issue of affordability comes under debate in questioning the very rationale of value-based pricing (VBP) of drugs.^4^ Relatedly, the Dutch case on pharmacist compounding features scholarly work co-authored by people at the Dutch Medicine for Society platform that outlines a “cost-based price calculation”, taking further steps in questioning pricing rationales and offering alternatives [63].

## Summary & discussion – addressing availability, accessibility and affordability through SPINs

Challenges in pharmaceutical innovation for rare diseases are not limited to upstream research and development; rather, downstream regulatory, HTA, and coverage dynamics are also at play. We have shown that SPINs may intervene in many stages, processes, and institutions involved in the production, regulation, and access to treatments, indicating that change is underway in the broader innovation system through which treatments for rare diseases are researched, developed, regulated, accessed, and used. These changes work to addresses challenges associated with the availability, accessibility, and affordability of treatments for rare diseases.

### SPINs address availability of treatments for rare diseases by diversifying players and roles in innovation

Public and charity-funded research organizations play a large role in drug discovery but struggle to bring medicinal products on the market [64]: the private sector often decides which innovations to push forward, driven by projected return on investment [31]. Despite significant promise of innovations like gene therapy to treat rare diseases, several programs have recently been abandoned due to escalating costs [65].

Many of our case studies demonstrate that SPIN partnerships extend beyond *just* public and private institutions to include up to seven ‘Ps’: some SPINs are, in fact, Public-Private-Patient-Physician-Philanthropic-Payer-Partnerships. Through these more diverse forms of collaboration, as in cases like Lumevoq©, Patient One, and the LIDOPAN study, inroads can be made into the lack of available treatments because there are more actors involved in deciding what gets developed and why it gets developed (i.e., not just for profit).

Further, availability issues are addressed through changes in the roles these actors play within collaborations and through changes in the substantive issues on which collaboration takes place. Upstream research and innovation work have been shown to be redistributed in all the French cases involving the AFM as this patient organization takes on roles conventionally held by biotech companies or university research centres. Decisions not only on what to develop but how to license products are now the prerogative of partnerships, thereby again widening the prospects of available treatments.

Finally, we see new tools applied to further enhance these collaborations. The case of G4RD demonstrates that more open and flexible public data infrastructures that are based on conditions (rather than treatments) allow for a better understanding of the biological basis of diseases, and wider sharing of that understanding.^5^ Tools that are more open, accessible, and premised on governed sharing may support the development of a broader array of treatments than registries focused on one specific treatment to serve regulatory approval purposes. In sum, by broadening the stakeholders, their roles, and the tools involved in innovation, SPINs contribute to redefining and addressing problems surrounding the lack of availability and how to deal with them.

### SPINs facilitate and speed up access to treatments by way of data

SPINs address access problems through new ways of articulating and gathering data on diagnosis, clinical efficacy, efficiency of the treatment, and real-world data, most notably for HTA purposes. The CUREiHUS, CFDI and the Brazilian RSA cases have shown how data is produced in ways outside of ‘gold standard’ clinical trials, and by actors outside of the pharmaceutical industry, to rescue treatments that would otherwise not be covered. These shifts in data gathering and sharing are a part of a broader trend in pharmaceutical innovation, which includes the production and use of real-world data (RWD) and real-world evidence (RWE) for rare and nonrare diseases [69].^6^ We see SPINs are often leading the way in the RWD/RWE movement. As such, SPINs are moving the production of data and evidence out of the exclusive hands of companies/promotors of clinical trials while providing new routes for patients to access effective treatments in faster, more localized, and potentially more financially sustainable ways. Our case findings are supported by other initiatives beyond our study scope that are simultaneously developing pathways for using new sources and types of data for regulatory approval.^7^

Pivotal in these data-related changes is the role of the clinical researcher. SPINs require skilled personnel that bridge the boundary between research and care, especially since many treatments are advanced, even experimental therapies. Next to these hybrid skills, clinical researchers are often the first point of contact for patients, will advocate for them, and are best positioned to see the impact of treatment on patients (if any) and analyse results accordingly. This makes them key players in the data collection, analysis, and accelerated pathway processes. We have also seen how clinical researchers are often well versed in the *realpolitik* of healthcare and pharmaceutical budgeting and allocations, which means they can establish links between data collection and concrete, novel accessibility instruments like start and stop criteria (e.g., the CUREiHUS case). Consequently, their involvement is of critical value in facilitating access to treatments, and grants SPINs a great deal of legitimacy [75].

### SPINs create new pathways to more affordable treatments

Finally, our case studies show that alternative pathways are emerging that provide access to treatments for rare diseases and do so at more affordable prices. Diverse actors conventionally outside of treatment production and manufacturing processes are becoming involved in producing more affordable medicines. The Dutch case of magistral preparation of treatments by pharmacists worked to make treatments available for patients, rescued certain medicines that have received negative HTA coverage advice, and challenged the high prices of certain products. Our SPIN cases also include public sector and in-house hospital manufacturing of CAR T-cell therapies by university researchers in Canada and the Netherlands. Taken together, these cases demonstrate the viability of treatment manufacturing and provision outside of pharmaceutical industry domains.^8^ While data is still forthcoming as to the extent to which these treatments are more affordable than their commercial counterparts, public sector manufacturing does remove pressure of price maximization in the name of profits and shareholder returns.

Those initiatives – together with the Brazilian PDP, the Dutch myTomorrows case, and all French cases involving the AFM – have shown new pathways to treatment by bringing treatment closer to the patients, and to a wider range of patients. Not only does bringing treatments closer to the patients stands to make them more attainable for patients, but also prospectively more affordable to payers.

## Conclusions, recommendations & future work

Many of our SPIN case studies have taken the form of projects or one-off initiatives designed to address a specific need or stress-point in the innovation system. We have seen such projects and experiments achieve success, of course, though, this is not the entire story. Below we highlight a few limitations to our study, challenges to SPINs more generally, and recommendations for moving them forward in research and practice.

### Limitations

From the outset, our focus has been on the prospects of social forms of *pharmaceutical* innovation. We recognize that these do not exist in a (clinical) care vacuum. Understanding the prospects for SPINs and addressing unmet needs in rare disease patients also requires paying attention to other aspects, such as the role of diagnostics, formal and informal care, support for carers, special training of doctors and clinical specialists (e.g., to recognize rare diseases, to appreciate HTA dynamics, and even for difficult ethical discussions around stopping treatment, to name a few). Even in a future that sees the dominant model of pharmaceutical innovation existing alongside SPIN models, many patients suffering from rare diseases will never see effective treatment. Consequently, time, energy, effort, and resources must continue to be invested in pre-clinical research for understanding the natural history of diseases, their genomic, genetic, and epigenetic bases, as well as forms of care and interventions that aid patients and carers which are not pharmaceutically derived.

While attention and resources are required for non-pharmaceutical interventions for rare disease patients, critical analysis is also essential for interventions within pharmaceutical innovation that may appear to be like SPIN. For instance, initiatives that provide accelerated market access or “adaptive pathways” for companies have come under criticism [77]. A recent study found that significant proportion of drugs that received American or European accelerated approval or conditional marketing authorization did not add high therapeutic value [78], and often subsequent data of treatment effectiveness did not follow after conditional approval [78, 79]. Mechanisms are required to remove treatments from market if adequate evidence is not forthcoming, and/or to share the cost of treatment while that data is being collected. In line with this critical reflection, we recognise that our focus on bio-pharma innovation reflects prioritization on high technology and advanced cell and gene-based therapies. However, we have seen that such priorities are disproportionally supported by groups and interests located in wealthy countries. Critically, such high-tech treatments are often embroiled with the innovation agendas of those wealthy countries [80, 81]. Further attention needs to be paid to equity challenges of availability, access, and affordability around the globe that have not been tackled in this project [82], to intellectual property, and its role there within [83, 84]. At the same time, we recognize that it is precisely these emerging high-tech solutions that are associated with possibilities of decentralized innovation and public sector manufacturing that stand to make treatments more accessible and affordable [85].

We have shown that some SPINs benefit especially from working with low-tech, easy-to-manufacture medicines or off-patent ones. Frugal innovation might be an additional solution if manufacturing processes can be become standardized, and drug repurposing and off-label use has demonstrated significant promise in rare diseases [86, 87], and particularly in the treatment of rare cancers [88]. In line with our analysis of SPINs, a key role stands to be played by academia in such initiatives [89], as well as multi-sectoral initiatives, like the European repurposing innovation platform REMEDi4ALL, primarily motivated by providing patients therapeutic options and not necessarily by prospective profits [90].

While we have shown that SPINs offer promise for resolving several problems in innovation in the area of rare diseases, it is vital to stress they are not ready-made solutions that can be taken off the shelf and applied anywhere. Furthermore, while some initiatives may be well established or taken for granted in some jurisdictions, those same initiatives might be considered innovative or unfeasible in other countries. We stress the importance of particular national contexts and institutions in our first recommendation below, and caution should be heeded against ‘me-too’ SPINs, or ‘plug-and-play’ attempts to replicate a SPIN initiative from one constituency to another.

### Recommendations for SPIN Strategy & Future Research

#### Local solutions are needed

Significant variation exists across institutions at the national level. Consequently, we argue that SPINs should be developed as specific solutions to the particulars of national priorities, problems, and framing, as well as the care, rare disease and innovation agendas and institutions that characterise respective countries. Local knowledge and expertise is paramount in crafting SPINs. A wide range of options exists in terms of addressing availability, accessibility, and affordability problems, but not everything is efficient, or even possible, everywhere. The SPINs we advocate for are purpose-built and tailor-made solutions to carefully understood specific problems [91]. A framing of SPIN that is informed by our interdisciplinary field of Science and Technology Studies (STS) helps to explain how and why SPIN cases have emerged out of a particular national context, and what may or may not be feasible elsewhere – and why. Local expertise and STS sensitivities may help with de-contextualizing and re-contextualizing processes to develop fit-for-purpose SPINs that address specific patients’ needs. Making a solution ready for another context entails at least five important points: (1) understanding local contexts, (2) improving the solution itself, (3) tailoring the solution to local contexts, (4) destabilizing existing solutions, and (5) embedding the solution in institutions and value chains (or change them) [92].

#### SPINs require a strategic and systemic approach

Developing these tailor-made, localized SPINs involve attending to the complex environment in which they must operate: the entire local innovation system. We have argued that innovation is not limited to upstream R&D for the develop new treatments, but mid- and down-stream issues of manufacturing, regulation, HTA, and licensing are also part of the innovation system that need reconsidering in light of availability, accessibility, and affordability problems. Through our case studies, we have shown that SPINs span and may disrupt different stages of the innovation lifecycle. Understanding, supporting, and implementing SPINs – and future work that seeks to do so – requires a systemic perspective that appreciates complexity, is interdisciplinary, and recognizes the interconnections between innovation system components [93, 94]. Acknowledging complexity and interconnections within the pharmaceutical innovation system is crucial as a SPIN initiative in one area (e.g., an access agreement) is likely to call for changes in other areas (e.g., in data collection and pricing).

#### Create a more explicit and comprehensive agenda for data governance, access, and ownership

Our research has shown that SPINs are highly data-driven. Like all pharmaceutical innovation, it requires the collection and sharing of data for discovery of aetiology to diagnosis, identification of treatment targets, to the development of treatment, and carrying out of trials, to regulatory and HTA decision-making processes: data plays a crucial role throughout. Not only are data infrastructures needed for all of this, but careful consideration needs to be given to the creation of publicly owned and operated infrastructures with carefully crafted governance and access rules. Furthermore, having such data and data infrastructures within the public domain means that data can be openly shared, or accessed through benefit sharing agreements, and used for a range of purposes rather than solely regulatory approval processes. Broad public infrastructures stand to reduce the costs of the next line of diagnostics and treatments once that initial outlay has been made. Critically, an eye needs to be kept on maintenance and long-term sustainability of such data infrastructures [95], and well-trained information/data specialists are needed to collect, input, and steward data, as such data-driven activities stand to overburden patients and clinical research specialists.

#### Build and test more comprehensive and inclusive economic models

Given that data is produced in clinical settings, through publicly funded research, and by patients seeking to receive publicly funded treatments, it is critical that the products of that data are accessible to patients and at a cost that is sustainable to healthcare systems. Situations where the public pays twice (i.e., once for the research and development needed, and then again with high prices for the treatment itself), like in the case of Zolgensma^®^ in France, must be avoided. Future research can draw on considerable scholarship and real-world examples of access and benefit sharing agreements in biobanking [96], and from painful lessons learned from public investments in underwriting the costs of developing, manufacturing and distributing the COVID-19 mRNA vaccines while private firms captured most of the profit [97]. If a desire exists to create SPINs, develop a more systematic approach that regularizes them, and/or work to ensure that they are not captured by market forces, then new economic models will also have to be explored that sustain such future initiatives. Importantly, these need not be only formal commercialization strategies [98]. Such new models should come with a reframing and rebalancing of the associated costs (and transparency of costs), risk-reward balance, and related responsibilities. They may be especially appropriate in the ultra-rare disease space, as incentives are lacking for the private sector to develop treatment for very, very few patients. Finally, significant pressure is mounting for increased transparency in R&D costs and pricing within the pharmaceutical system [99], and it is critical that SPINs build economic viability in an open and accountable manner.

### Closing words

We hold that future drug development and diffusion needs to become more just and equitable for all [100], and in line with the Sustainable Development Goals (SDGs). Achieving SDG3, that ensuring healthy lives and promoting well-being for all at all ages [101], is severely challenged by significant flaws in the current pharmaceutical innovation system, while societal pressures to develop and diffuse therapies for unmet medical needs is increasing. Availability, accessibility, and affordability of these and other treatments needs to be ensured. This paper has provided interesting insights based on current, real-world cases of how this could be achieved in the future. While still niche initiatives within the grand scheme of pharmaceutical innovation, other examples of SPIN exist around the world and continue to emerge. We restate the invitation from our position paper on SPIN in this journal [30] for future collaborations to advance and critically analyse SPINs, and we continue to offer our interdisciplinary expertise and perspectives in doing so.

## Data Availability

Materials used to conduct semi-structured interviews is available upon request. Interview data is not available to protect identify of respondents.

## Footnotes

1 The percentages of conditions that have treatments vary widely around the world. While the European Union does share regulatory mechanisms for market authorization that does not mean that all member states, and across the European Union, have uniform access to treatment.

2 These high prices are also considered by some commentators to be the result of the monopolization of profits of innovation processes through patents [14, 15]. In such a system, profits are generated through patent rents, which are then distributed to shareholders and used for future mergers and acquisitions rather than reinvested into research [16].

3 The technology transfer involved in *Parcerias para o Desenvolvimento Produtivo* / Partnerships for Productive Development is distinct from how the WHO characterises Product Development Partnerships, which they define as “public–private partnerships established to develop and provide access to new health products – especially vaccines, therapeutics, and diagnostics – for poverty-related and neglected diseases. PDPs are non-profit-making and typically funded by public and philanthropic organizations”. Confusingly, both are referred to as PDPs.

4 While VBP is meant to determine the price of a treatment based on its future benefits to patients and health systems, patient organizations like the French AFM and NGOs claimed that the prices of treatments – in this case Zolgensma© – should instead consider R&D and production costs for two main reasons. First, because early research was conducted by the French public research institution CNRS in collaboration with Généthon (i.e., AFM’s lab) and benefited from French public money and money donated by the French population through the Téléthon (i.e., AFM’s fundraising event). Second, because VBP is future-oriented and therefore lends itself to price speculations that threaten the sustainability of health systems. The subsequent high price of Zolgensma provoked resentment in France [60], while the Netherlands has rationed provision to a narrower patient population [61]. Though Zolgensma© has been approved in 51 countries and has been used to treat approximately 3,700 patients, only 35 countries have reimbursedthecosts [62].

5 Other such tools also exist in the form of 24 European Reference Networks (ERNs) co-funded by the European Commission to develop their research capabilities, including the construction of patient registries [66], and the European Rare Disease Registry Infrastructure (ERDRI) renders rare disease registries’ data searchable and findable [67]. The North American Organization for Rare Disorders (NORD) has created a natural histories patient registry platform as part of the IAMRARE Program with “extensive input from FDA, NIH, patients, organizations and experts in the field to inform and shape medical research and translational science for rare diseases by launching high-quality, customized registries to collect the data needed to define the natural progression of their disease – ultimately advancing product development” [68].

6 Also part of this broader trend and transformation, but not covered here, are other forms of trials that depart from the double blind, placebo-controlled trials [70], such as n-of-1 trials [71–73] and basket trials [11]. To that end, the non-profit Professional Society for Health Economics and Outcomes Research (better known as ISPOR) has made recommendations that different kinds of evidence in the form of patient-reported outcomes (PROs) be elaborated with patient organizations and observer-reported outcomes (OROs) and be included in clinical trials for treatments for rare diseases [74].

7 This includes the Dutch Orphan Drug Access Protocol that provides access to a small number of drugs for rare diseases “simultaneously gather additional information about long-term effectiveness and safety of the drugs, while arranging its accessibility and pricing” [50]. Similarly, the International Horizon Scanning Initiative (IHSI) exists as a platform that “provides data that empowers political decision-makers and payer organisation negotiators to drive for better pricing in medicinal products” [51]. Included within the IHSI is a High Impact Reports (HIRs) that “analyse and evaluate specific information sets from the database to highlight pharmaceuticals with a high potential to cause significant impact” [51]. Numerous collaborations have emerged for collecting and using new forms of data for the assessment of the cost-effectiveness of new medicines such as the collaboration between Canada-NICE-Australia-Scotland, Wales [54]. Similarly, the BeNeLuxA initiative on pharmaceutical health policy facilitates the re-use of HTA reports from one jurisdiction to another, as well as the joint writing of an HTA reports, the mutual recognition of HTA reports, and the joint negotiation purchasing agreements [55].

8 These findings are supported by similar initiatives taking place in other jurisdictions, such as Spanish clinical researchers that have pioneered the use of regulatory feature known as a “hospital exemption” that allows for the development and delivery of CAR T-cell treatment locally within a hospital under very precise circumstances after safety and efficacy of treatment has been proven through trials. The national competent authorities (rather than the supra-national European Medicines Agency in this case) allow for the provision of the advanced therapies under special conditions: (1) it is limited to each member state of the European Union (EU) under specific regulations; (2) it is limited to the authorized hospital, developing mainly the ATMP; and. (3) it is used under the responsibility of that hospital’s staff members [76]. This is neither the kind of clinical trial model discussed above nor a product that has received market authorization, but nevertheless a new pathway for patients to access treatment that has not existed in the dominant biopharmaceutical innovation model.
